# Impactful publications of critical care medicine research in China: A bibliometric analysis

**DOI:** 10.3389/fmed.2022.974025

**Published:** 2022-10-18

**Authors:** Wei Qiang, Chuan Xiao, Zhe Li, Li Yang, Feng Shen, Lin Zeng, Penglin Ma

**Affiliations:** ^1^Department of Library, Guizhou Medical University, Guiyang, China; ^2^Department of Intensive Care Unit, The Affiliated Hospital of Guizhou Medical University, Guiyang, China; ^3^Department of Critical Care Medicine, Renji Hospital, School of Medicine, Shanghai Jiao Tong University, Shanghai, China; ^4^Research Center of Clinical Epidemiology, Peking University Third Hospital, Beijing, China; ^5^Department of Critical Care Medicine, Guiqian International General Hospital, Guiyang, China

**Keywords:** China, research, impactful publications, critical care medicine, factors, bibliometric analysis

## Abstract

**Background:**

Although publications have been increasing rapidly, the research quality has yet to improve in the field of critical care medicine (CCM) in China. This study aimed at investigating the current status of and the influential factors for impactful publications in CCM research by Chinese authors.

**Methods:**

Publications by authors with the affiliation of critical care medicine department or intensive care unit (CCM/ICU) in Chinese as well as American hospitals from 2001 to 2020 were retrieved from the Web of Science Core Collection (WoSCC) database for this bibliometric analysis. Moreover, statistical analyses to test factors affecting impactful publications by Chinese authors were performed.

**Results:**

Of 13,487 articles retrieved by this search strategy, 6,622 were published by Chinese authors as first or corresponding authors. The annual publications by Chinese authors have been rapidly increasing from 2001 to 2020, and so did the citations to these articles. However, the proportion in the world of publications by Chinese authors was much less than that by American authors each year [*M* (IQR): 1.85 (9.592) vs. 27.77 (7.3), *p* < 0.001]. In addition, impactful articles were significantly less published by Chinese than by American authors, including articles either in journals with a high impact factor (*p* < 0.001) or in the top 10 journals in the field of CCM (5.4 vs 13.4%, *p* < 0.001), and articles with high citation frequency as well (*p* < 0.001). Moreover, the percentage of impactful publications by Chinese authors was likely associated with academic background and regions of the author's affiliations, funds support, public health events of COVID-19, and collaboration between authors.

**Conclusion:**

Our results demonstrated that CCM research in China grew rapidly in the recent 20 years. However, the impactful publications remained limited, largely owing to the shortage of comprehensive research training, inactive collaboration, and underfunded CCM research.

## Introduction

Critical care medicine (CCM) in China was seen to make great progress in the past two decades (1). It plays an important role not only in the management of critical illness in hospitals but also in increasing actions of public health emergencies and natural disasters. However, the achievement of research was not consistent with clinical practice in the field of CCM in China. Based on bibliometric analysis, Li et al. (2) reported that the number of publications on CCM was much less in China than that in the United States and other developed countries from 2000 to 2010. Moreover, the research with high quality were mostly concentrated in Taiwan and Hong Kong. In fact, the majority of articles in this field from mainland China were less impactful during this period (3).

To promote the scientific research in CCM, experts from the Chinese Society of Critical Care Medicine established the China Critical Care Clinical Trials Group (CCCCTG), comprising intensivists from 24 ICUs from 21 provinces in China, which joined the Global Sepsis Alliance (GSA) in 2010. In addition, there were more and more activities specific to scientific research training, for example, the Conference of Critical Care Research Forum (CCCRF), Salon for Young Critical Care Investigators, and Critical Care Research Campaign, etc (4). Accordingly, the number of publications on CCM from China has been increasing rapidly over the last decade (5–8). Meanwhile, the research quality has yet to improve. An updated bibliometric analysis showed that China contributed only 1% of the top 2,000 highly cited articles on critical care, as of 13 February 2018 (9). In addition, there never was an article on CCM from China with annual citations over 100 before 2018 (10). These data suggest that problems remain in promoting the quality of research on CCM in China. Notably, the barriers were under-investigated. Therefore, this study aimed at investigating the current status of and the influential factors for impactful publications in CCM research from 2001 to 2020 by Chinese authors, who reported the affiliation of Critical Care Medicine department or intensive care unit (CCM/ICU) in Chinese hospitals, through a bibliometric and visualized analysis.

## Methods

### Data sources and search strategies

Web of Science Core Collection (WoSCC) database is one of the most comprehensive, systematic, and authoritative databases, which has been successfully used for bibliometric analysis (11, 12). Publications by authors reporting the affiliation of CCM/ICU in Chinese hospitals from 2001 to 2020 were retrieved for this bibliometric analysis. The search strategy was “Address: (Chinese OR China OR CN) AND (Intense Care Unit OR Crit Care OR ICU OR intensive care OR critical care) NOT Address: (Respiratory OR Pulmonary OR PCCM).” The data set retrieved from the WoSCC database was transformed into an Excel version. The collected articles were further screened by the first or corresponding authors who reported the affiliation of CCM/ICU in Chinese hospitals. Being a comparator, data regarding publications from CCM/ICU in American hospitals were collected by the same search strategy, but “American OR America OR US” replaced “Chinese OR China OR CN.” Time windows were unified as “1 January 2001 to 31 December 2020”; and there were no language or article type restrictions. All data were collected online on 1 May 2022 and no ethical proof was required.

### Data collection

Data regarding publications retrieved from WoSCC included title, keywords, authors, affiliations and regions, journal, date of publication, funding, citations, etc. Data were extracted by two authors (QW and ZL) independently and the agreement of the results was 98%, showing significant consistency. All data were saved in a text or excel format for further analysis.

### Bibliometric analysis

All downloaded documents were imported to the Web of Science-Incites Research Performance Analysis Platform (WoS-Incites, https://incites.clarivate.com/), VOSviewer (version 1.6.15), and Microsoft Excel 2019. WoS-Incites were used to analyze the number of publications, impact factors of the journals, citation frequency, characteristics of the authors, and their affiliations. VOS viewer 1.6.15 (Leiden University, Leiden, The Netherlands) was used to analyze and visualize co-authorship of authors, institutes, countries, and co-occurrence analysis of keywords (13). Microsoft Excel 2019 was used to diagrammatize results from WoS-Incites (14).

### Statistical analysis

Continuous variables were expressed as mean ± standard deviation (mean ± SD) or median [interquartile range; *M* (IQR)] depending on whether they followed a normal distribution. Differences between groups were compared by Student's *t*-test or the Wilcoxon rank sum test based on data distribution. Categorical variables were described using cases and percentages or proportions. And differences between groups were compared by the chi-square test or Fisher's exact probability method. Two-sided *p-*values < 0.05 were considered statistically significant.

## Results

### Publications and citations

#### Publications

**Figure 1:**
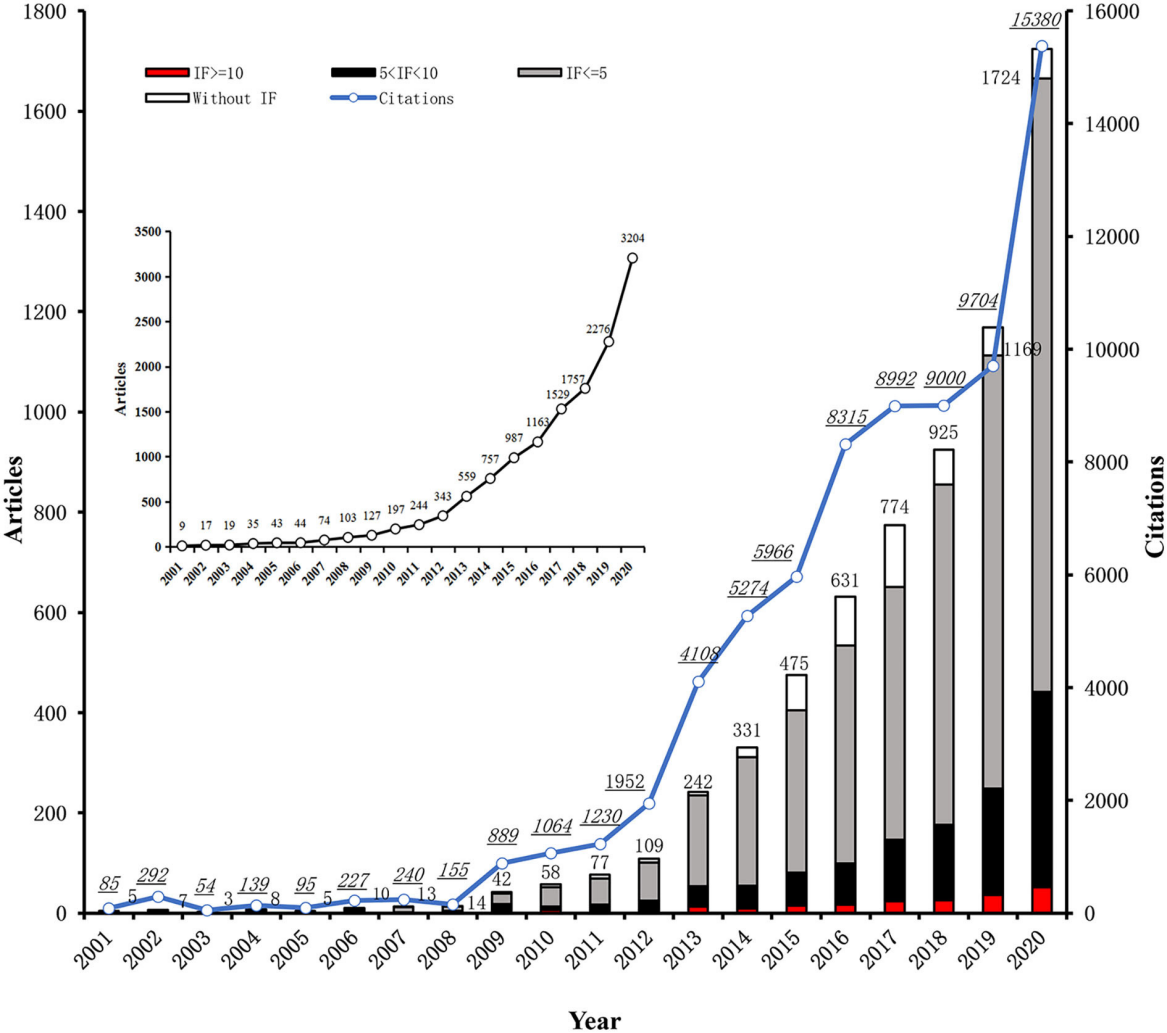
Annual publications and the cumulative citations from 2001 to 2020. The X-axis represented each year from 2001 to 2020, the Y-axis represented the number of annual publications (bar) and the cumulative citations (blue line) to all articles till the year published by first and corresponding authors from affiliations of Critical Care Medicine (CCM) department or intensive care unit (ICU) in Chinese hospitals. These annual publications were stratified by the impact factor (IF) of the journals publishing these articles, marked on red (IF ≥ 10), black (5 < IF < 10), gray (IF ≤ 5) and white (without IF) as well. The inner figure showed the trend of annual publications with one author at least on author list from affiliations of CCM department or ICU in Chinese hospitals.

There were 13,487 articles published in international peer-reviewed journals listed on the Science Citation Index (SCI) from 2001 to 2020 reporting one author at least with the affiliation of CCM/ICU in Chinese hospitals. The number of annual publications was over 100 in 2008 and has been rapidly increasing since 2008 (Figure 1 inner).

Out of the 13,487 publications, 6,622 were further retrieved by the first or corresponding author who reported the affiliation of CCM /ICU in Chinese hospitals (Figure 1). Stratified with the impact factors (IFs), 531 (8.02%), 4,685 (70.75%), 1,192 (18.00%), and 214 (3.23%) out of 6,622 articles were published in journals without IF, IF ≤ 5, IF between 5–10 (5 < IF < 10) and IF ≥ 10, respectively (Supplementary Figure S1). Notably, the number of annual publications in journals with IF > 5 would not exceed 100 until 2017, while publications were over 50 in journals with IF ≥ 10 till 2020 (Figure 1).

#### Publications by Chinese vs. American authors

The proportion of publications in the world on CCM research from 2001 to 2020 by Chinese authors was much less than that by American authors [*M* (IQR): 1.9 (0.4, 10.0) vs 27.8 (25.6, 32.9), *p* < 0.001, Table 1]. However, the proportions contributed by Chinese authors increased yearly, while a decreased trend was found in that by American authors in this study period (Supplementary Table S1). As shown in Supplementary Figure S2, the proportion of publications by American authors always ranked first in the world each year from 2001 to 2020. The ranking of publications by Chinese authors has entered the top 10 since 2012 (rank ninth) and kept the second place since 2014. Significantly, the number and percentage (the number/the total) of publications in the top 10 high impactful journals in the field of CCM (including NEJM; JAMA, BMJ, Am J Resp Crit Care Med, Intensive Care Med, Critical Care Med, Ann Intensive Care, the detailed data are shown in Supplementary Table S2) were also much less by Chinese authors than by American authors in these two decades [358 (5.4%) vs 3,060 (13.4%), *p* < 0.001, Table 1].

**Table 1:** Publications and the citations by Chinese vs. American authors from 2001 to 2020.

	**By Chinese authors**	**By American authors**	***p*** **-Value**
Publications in total	6,622	22,819	—
**Publications in journals with**, ***n*** **(%)**			
IF < 5	4,669 (70.5)	13,116 (57.5)	
IF ≥ 5	1,953 (29.5)	9,703 (42.5)	<0.001
IF ≥ 10	608 (9.2)	3,066 (13.4)	
Publications in top 10 impactful journals linked to CCM^†^, *n* (%)	358 (5.4)	3,060 (13.4)	<0.001
Yearly proportions of publication in the world (%)^#^, *M* (IQR)	1.9 (0.4, 10.0)	27.8 (25.6, 32.9)	<0.001
**The top 20 keywords in publications**, ***n*** **(%)**			
Categorized to basic research	7 (35.0)	0 (0.0)	0.008
Categorized to clinical research	13 (65.0)	20 (100.0)	
Yearly citation frequency (yearly citations/articles, %), *M* (IQR)	17.0 (11.9, 18.4)	27.8 (15.9, 36.9)	0.012
Individual citation frequency in WOSCC^††^, *M* (IQR)	5.0 (2.0, 14.0)	8.0 (1.0, 24.0)	<0.001
Highly citated articles^*^, ***n*** **(%)**			
In top 10	1 (10.0)	3 (30.0)	<0.001
In top 100	3 (3.0)	55 (55.0)	
In top 1,000	21 (2.1)	660 (66.0)	

† The top 10 impactful journals linked to CCM include NEJM (NEW ENGLAND JOURNAL OF MEDICINE); JAMA (JOURNAL OF THE AMERICAN MEDICAL ASSOCIATION); BMJ (British Medical Journal); Am J Resp Crit Care Med (AMERICAN JOURNAL OF RESPIRATORY AND CRITICAL CARE MEDICINE); Intensive Care Med(INTENSIVE CARE MEDICINE); Critical Care Med (CRITICAL CARE MEDICINE; Ann Intensive Care (ANNALS OF INTENSIVE CARE), the detailed data regarding publications are shown in Supplementary Table S1.

# Yearly proportions of publication: the proportions of publications in the world each year from 2001 to 2020 by Chinese vs. American authors on CCM researches, the detailed data are shown in Supplementary Table S1.

†† Individual citation frequency in WOSCC: Citations of each publication in the database of WOSCC (Web of Science Core Collection).

* The highly cited articles: the top 10, 100 and 1,000 highly cited articles in the world.

### The keywords in publications

**Figure 2:**
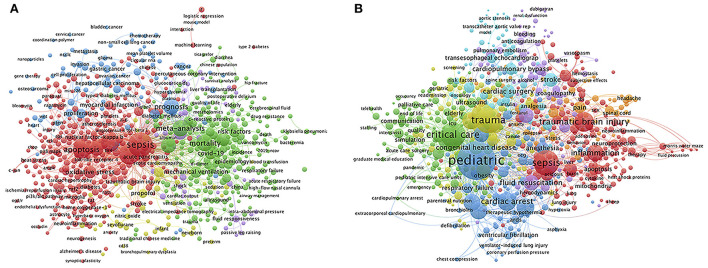
Visualization map of Keywords co-occurrence network in publications on Critical Care Medicine research. Keywords co-occurrence network in articles published by authors from affiliations of CCM or ICU in Chinese **(A)** vs American **(B)** hospitals from 2001 to 2020 were mapped. The size of the circles indicated the co-occurrence frequency of keywords. The color of each circle indicated clusters, which was a set of keywords calculated in the co-occurrence network as a community. The connecting lines indicated co-occurrence of the 2 keywords at both ends. The thickness of lines between circles indicated strength of linkage calculated by the frequency of co-occurrence.

A total number of 10,980 and 21,689 keywords were identified from 6,622 and 22,819 publications by first and corresponding authors with affiliations of CCM/ICU in Chinese and American hospitals, respectively. The top 724 keywords with co-occurrence frequency equal to or over five were selected for co-occurrence network and overlay analysis. The occurrence frequency of keyword was displayed in circle size, as shown in Figures 2A,B. It was shown that “Sepsis” was only the same one out of the top five keywords (ranked by the occurrence frequency) in publications by either Chinese authors [“Sepsis” (642), “Acute lung injury” (412), “Inflammation” (289), “Apoptosis” (268), “Mortality” (232)] or America authors [“Pediatric” (1,402), “Trauma” (624), “Sepsis” (605), “Critical care” (584), “Intensive care unit” (546); Figures 2A,B, Supplementary Table S3], respectively. In addition, eight keywords including “Sepsis,” “Septic shock,” “Acute kidney injury,” Acute lung injury,” “Mechanical ventilation,” “Inflammation,” “Mortality,” and “Intensive care unit” were shared in the top 20 keywords by both Chinese and American publications (Supplementary Table S3). Moreover, it was demonstrated that seven and 13 keywords of Chinese publications, in comparison with 0 and 20 keywords of American publications, were categorized as basic researches and clinical researches, respectively (*p* = 0.008, Table 1).

In addition, the overlay analysis of the keywords represented the trends of topics in Chinese and American publications between 2001 and 2020. The circles of keywords were marked on colors from blue to yellow to display the overlay visual map of the keywords over time, which was quantitively calculated by the average publication year of the articles in which the keyword appeared (Avg.pub.year) (15). It was demonstrated that the Avg.pub.year of “nuclear factor-kappa B (2014.29, Ranked eighth)” and “ischemia/reperfusion injury (2013.75, Ranked sixth)” in publications by Chinese authors were about 7–9 years delayed from that of the similar keywords “nuclear factor-kappa B (2005.44, Ranked first)” and “reperfusion (2006.10, Ranked fourth)” by authors from America among the top 10 earliest research topics (Supplementary Figures S3A,B, Supplementary Table S4). As shown in Supplementary Table S4, the latest research topics were similar in publications by authors from China and America, that mainly focused on COVID-19, and the Avg.pub.years of these hot topics were from 2019 to 2020.

#### Citations

The citations to articles published by the first or corresponding authors with the affiliation of CCM /ICU in Chinese hospitals each year from 2001 to 2020 are also shown in Figure 1. Similar to the trend of publications, the total citations to these articles kept a rapid growth year by year from 2012, despite a rollback in 2018 (Figure 1). Out of the top 10 highly cited articles by Chinese authors, only one was not related to COVID-19 as shown in Supplementary Table S5 (16). The total citations of the top 10 highly cited articles in the world ranged from 3,116 to 10,788 from 2001 to 2020 (Supplementary Table S5), of which there was only one article published by the Chinese author (17). Significantly, the percentage of articles published by Chinese authors was much lower than that of American authors in the top 10, 100, and 1,000 highly cited articles in the world, as shown in Table 1 (*p* < 0.001). Additionally, either the average citation frequency (citations/articles) in the two decades [*M* (IQR): 17.0 (11.9, 18.4) vs. 27.8 (15.9, 36.9), *p* = 0.012] or the individual citation frequency in WOSCC [citations of individual article; *M* (IQR): 5.0 (2.0, 14.0) vs. 8.0 (1.0, 24.0), *p* < 0.001] was significantly lower in Chinese authors publications (Table 1).

### Factors barred to or facilitated the impactful publications in Chinese CCM research

#### Academic background of the authors' affiliations

As shown in Supplementary Figure S4, 69.55% (491/706) of the first and corresponding authors reported affiliations of CCM/ ICU in Chinese hospitals with academic background, including 65.72 and 3.82% of them affiliated with university (or college) and research institutes, respectively. Meanwhile, only 18.84% of the authors served hospitals without academic background (i.e., hospitals not affiliated with any university, college, or research institutes). Significantly, the percentages of articles published in journals with IF ≥ 5 and IF ≥ 10 by authors from academic hospitals were significantly higher than that from non-academic hospitals (*p* < 0.001, Table 2).

**Table 2:** Factors affecting the impactful publications in CCM researches by Chinese authors from 2001 to 2020.

	**IF < 5**	**IF ≥ 5**	**IF ≥ 10**	***p*** **-Value**
Authors from academic hospital^*^, ***n*** **(%)**					
Yes (total = 5,406)	3,690 (68.3)	1,716 (31.7)	518 (9.6)	<0.001
No (total = 1,216)	980 (80.6)	236 (19.4)	90 (7.4)	
**Fund for publications^#^, ***n*** (%)**					
Yes (*n* = 2,307)	1,467 (63.6)	840 (36.4)	198 (8.6)	<0.001
No (*n* = 4,315)	3,203 (74.2)	1,112 (25.8)	410 (9.5)	
**Publications related to COVID-19^†^, ***n*** (%)**					
Yes (in 2020, *n* = 206)	81 (39.3)	125 (60.7)	71 (34.5)	<0.001
No (in 2020, *n* = 1,518)	1,058 (69.7)	460 (30.3)	118 (7.8)	
No (in 2019, *n* = 1,169)	920 (78.7)	249 (21.3)	36 (3.1)	

IF: impact factor of the journals, which published the articles.

* Academic hospital: the word “institute” or “college” or “university” was reported in the affiliation of the first or corresponding author.

# Fund: it was based on the declaration of the article.

† COVID-19: any keywords with regard to COVID-19 (including coronavirus disease, SARS-CoV-2, novel coronavirus pneumonia, etc.) was found in title/abstract.

#### Geographic distribution of publications by Chinese authors

**Figure 3:**
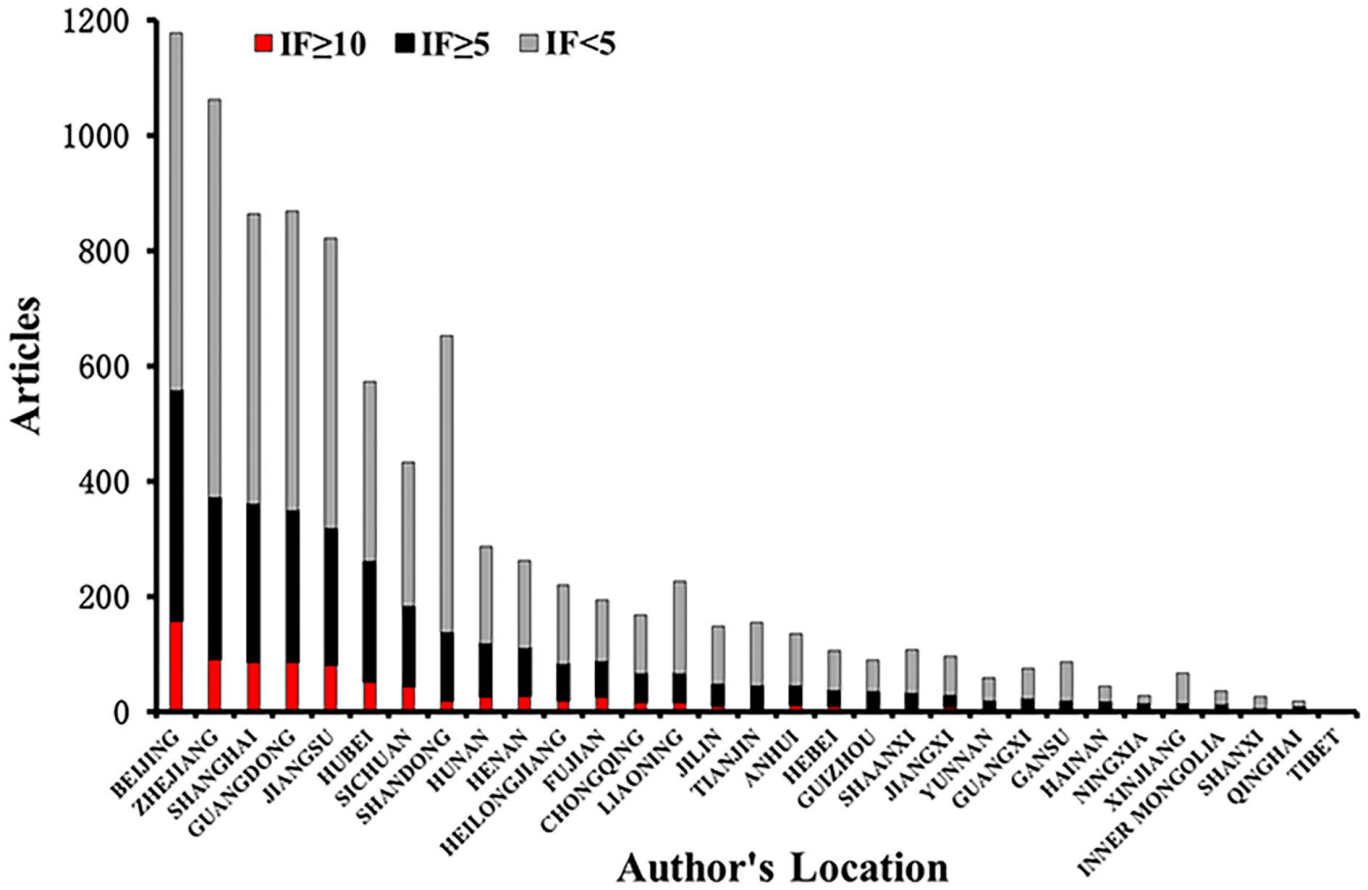
Geographical distribution of the publications by Chinese authors. The X-axis were the provinces/municipalities, where the hospitals of the first or corresponding authors of the publications were located. The Y-axis represented the number of their publications from 2001 to 2020, which were stratified by the impact factor (IF) of the journals publishing these articles, marked on red (IF ≥ 10), black (IF < 5), and gray (IF < 5) as well. A disequilibrium analysis of the distribution of impactful publications among regions from 2001 to 2020 were performed by using Fisher's exact probability analysis, and the result suggested that P < 0.001, which indicated the number of publications in different impact factor groups varied by regions.

The affiliations of 6,622 publications were distributed in 31 provinces/municipalities of China (Figure 3). Beijing was the only one out of the 31 provinces/municipalities with publications over 1,000. Meanwhile, there were 13 provinces/municipalities with publications <100 as shown in Figure 3. Significantly, <50 articles were published by authors from Hainan, Tibet, Qinghai, Inner Mongolia, Ningxia, and Shanxi province/municipality in these two decades.

By a Fisher's exact probability analysis, a significant disequilibrium was found in the distribution of the proportions of publications stratified with IF < 5, IF ≥ 5, and IF ≥ 10 in 31 provinces or municipalities of mainland China (the detailed data are shown in Supplementary Table S6).

#### COVID-19-related publications

There were 206 (11.94%) out of 1,724 publications focused on COVID-19 research in 2020. Compared with non–COVID-19 publications in 2020 and in 2019 as well, the percentages of COVID-19-related publications in impactful journals (i.e., IF ≥ 5 or IF ≥ 10) were significantly increased (*p* < 0.001, Table 2).

#### Funds supporting

Out of 6,622 publications by Chinese authors, 2,307 (34.84%) articles were reported with funds support. In comparison with the percentage of publications without fund, the percentage of those with funds supporting was significantly increased in journals with either IF ≥ 5 (36.41 vs. 25.77%) or IF ≥ 10 (8.58 vs. 9.50%; *p* < 0.001, Table 2).

#### Collaboration network analysis of the authors, institutes, and countries

**Figure 4:**
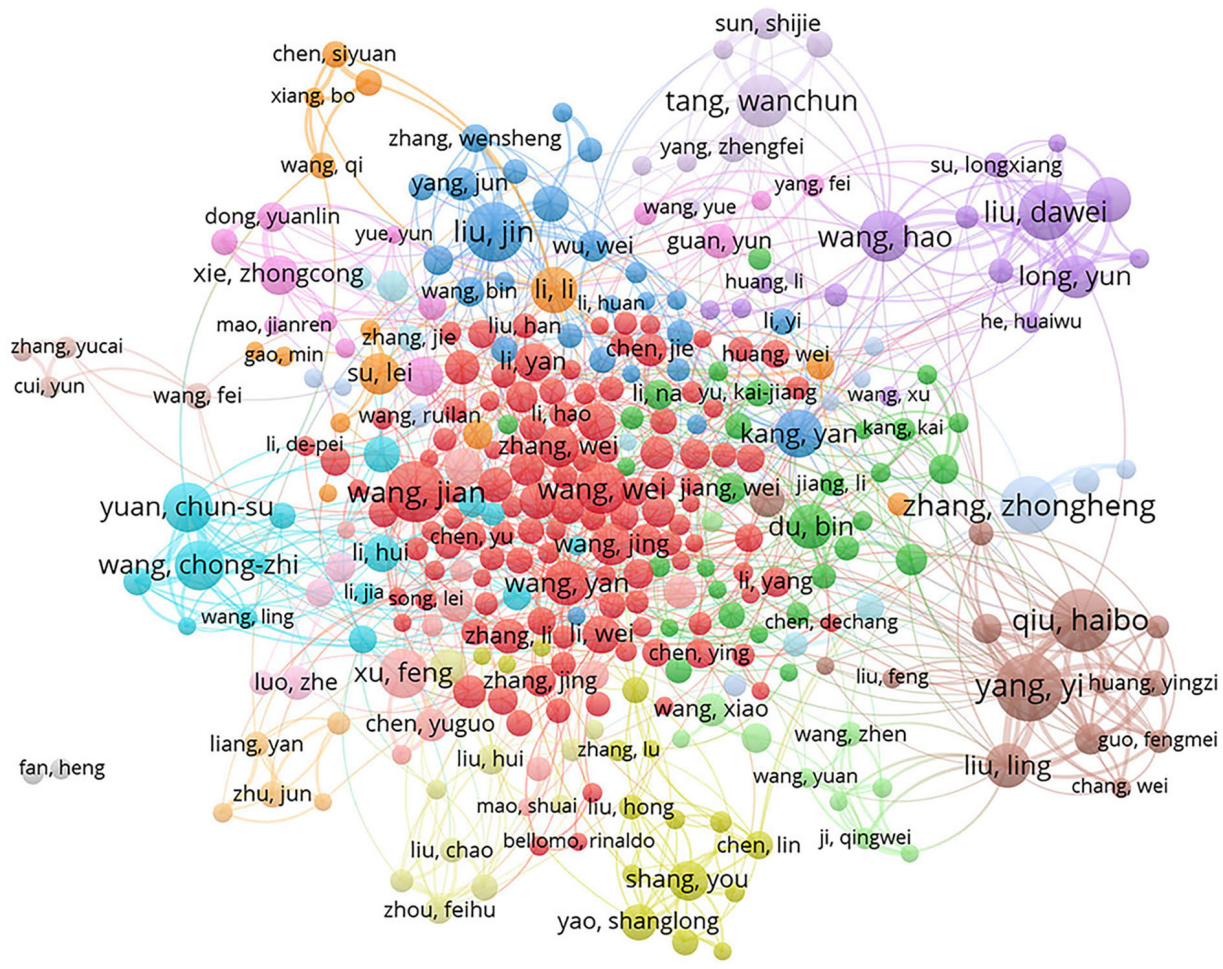
Cluster visualization map of the authors Co-authorship in research of Critical Care Medicine from 2001 to 2020. Each circle represented one author and circle size indicated number of his/her publications from 2001 to 2020. The lines between two circles indicated co-appearance of authors in an article. The thickness of lines indicated strength of linkage calculated by the number of publications. The color of each circle indicated cluster, which was a set of authors calculated in the co-authorship network as a community.

**Figure 5:**
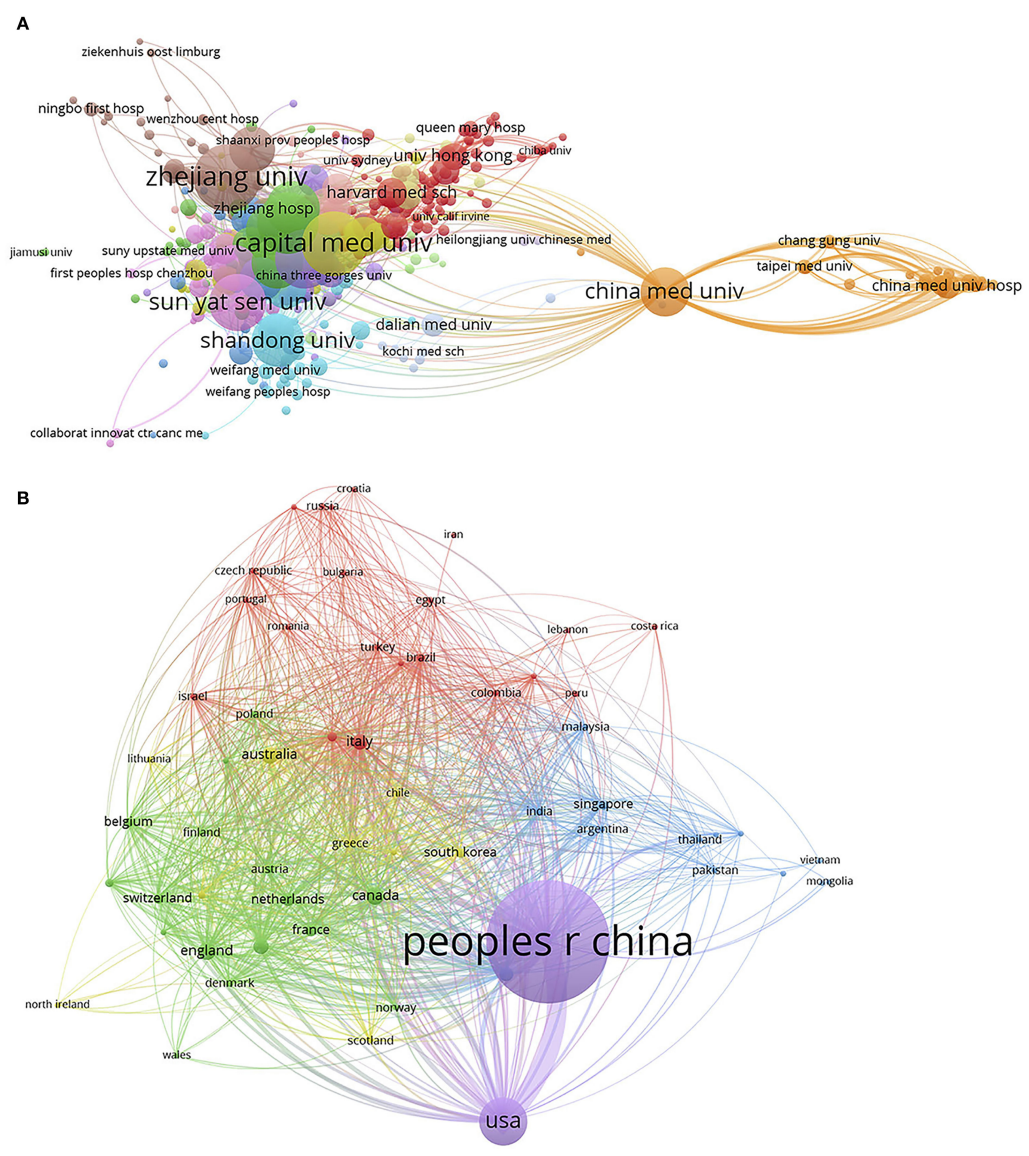
National and international Co-authorship network. Cluster regarding collaboration in Critical Care Medicine researches was mapped between authors from different affiliations of China (i.e national collaboration, **A**) or between authors from China and those from other countries (i.e international collaboration, **B**) from 2001 to 2020. Each circle indicated an affiliation of China, or one country. The circle size represented the number of publications and the lines between two circles indicated co-appearance of two affiliations or countries in one article. Color of each circle indicated the cluster. The thickness of lines indicated strength of linkage calculated by the number of publications co-authored by the different affiliations or countries.

The collaboration network visual map between the authors, the institutes, and the countries in the 13,487 articles was generated by VOS viewer (Figures 4, 5A,B). The total link strength was calculated on the number of publications co-authored by the authors, the institutes, and the countries. Of all 45,266 authors on the author list of the 13,487 articles, 342 who published 20 or more articles were analyzed. Ranked with the total link strength, the top three authors were “yang, yi” (419), “qiu, haibo” (413), and “liu, ling” (273), who come from the same affiliation, the Department of Critical Care Medicine, Nanjing Zhong da Hospital, School of Medicine, Southeast University, Nanjing. In addition, there were three other authors (“liu, dawei,” “long, yun,” and “wang, hao”) in the top 10 authors with the most collaborations came from the same affiliation too, The Department of Critical Care Medicine, Peking Union Medical College Hospital, as shown in Figure 4, Supplementary Table S7.

Out of the total 6,372 institutes of these authors, 398 published 10 or more articles. The Capital Medical University was the affiliation with the highest collaboration link strength (capital med univ, 1,162), followed by the China Medical University (China med univ, 1,072) and Shanghai Jiao Tong University (shanghai jiao tong univ, 1,037, Figure 5A, Supplementary Table S7). Moreover, authors from 61 countries collaborated with Chinese authors in five or more publications among the 13,487 articles. Authors from America (“usa”) collaborated with Chinese authors (“peoples r China”) most, followed by authors from “Italy,” “England,” and “Canada” (Figure 5B, Supplementary Table S7).

## Discussion

It was demonstrated that the publications by the first or corresponding authors with the affiliation of CCM/ICU in Chinese hospitals have been rapidly increasing from 2001 to 2020, and so did the citations to these articles (Figure 1). However, the proportion in the world of publications on CCM research by Chinese authors was much less than that by American authors each year (Table 1). In addition, the number and the percentage of impactful articles were significantly less published by Chinese than by American authors, including articles published in journals with a high impact factor (i.e., IF ≥ 5, IF ≥ 10), articles in the top 10 journals in the field of critical care medicine, and the high frequently cited articles as well (Table 1). Moreover, it was found that several factors likely affected the output of impactful publications in CCM researches by authors with the affiliation of CCM/ICU in Chinese hospitals, such as the academic background of authors affiliations, funds support, public health event of COVID-19, regions of author's affiliation and collaboration between authors (Table 2, Figure 4).

Previous studies suggested that several factors facilitate Chinese CCM research, including rapid economic growth, expansion of ICUs and intensive care practitioners (18), and responses to disasters such as SARS 2003, Wenchuan earthquake in 2008, the outbreak of COVID-19, etc. (1, 19, 20). Furthermore, it was demonstrated that the public health event of COVID-19 was associated with the production of higher impactful publications by Chinese authors in this study (Table 2). Meanwhile, the rapid increase of publication in CCM research in China could be also driven by the academic evaluation system in the past two decades largely. Although there have never been any officially issued rules, in fact, articles, awards, titles, degrees, and honors were highly weighted in the evaluation of an individual or team's competitiveness. Based on the regulations of most medical colleges or institutes, for instance, the candidates were not qualified to apply Doctor of Philosophy (PhD) or Medical Doctor (MD) degree until publishing one article at least in the SCI journal. Notably, reports of this evaluation would be closely tied with professional promotion and appointment. In this study, interestingly, several findings supported this approach, which was not evidenced in the previous studies (9, 10). First, hospitals where the most authors served (69.55%) were affiliated with academic institutes (Supplementary Figure S4). Few of them could be unaffected by this hidden regulation. Moreover, very few authors (18.8%) served nonacademic hospitals. According to the data from Beijing clinical quality control and improvement center for Critical Care Medicine, there are only 25 (29.8%) ICUs (including general, surgical, or medical ICUs) in hospitals with academic backgrounds among a total number of 84 grade II and III hospitals even in Beijing (unreported data). Second, over half of the articles (59.6%, 4,927/8,268) were published by authors from six out of 31 provinces/municipalities including Beijing, Zhejiang, Guangdong, Shanghai, Jiangsu, and Shandong (Figure 3), where medical colleges and research institutes were highly concentrated in China. Finally, the keywords of these articles were linked to lab research rather than clinical topics more frequently (Supplementary Table S3). These findings suggested that the majority of Chinese intensivists working in nonacademic hospitals have not successfully published articles in SCI journals during this period. There could be no argument about intensivists in academic hospitals getting better training and having a higher passion for scientific research. But, our results suggested that the research of Chinese intensivists is, partly at least, driven by the academic evaluation system rather than by their interests in questions arising through the day-to-day care of critically ill patients. Fortunately, a special notification was issued by the government for correcting the disadvantage of this evaluation system (https://news.sciencenet.cn/). Hopefully, the researches of Chinese intensivists will be conducted with the impetus to study questions arising through intensive caring. In this way, the production of Chinese intensivists' research will be not only rich but more impactful in future.

A comprehensive training in scientific research is the base for highly impactful publications. Our findings suggested that authors who got better research training probably, for instance, who served in hospitals with academic backgrounds and in cities with more medical colleges/universities/research institutes, be more likely to publish impactful articles (Table 2). To our knowledge, however, there was an acute shortage of training courses specific to critical care research in China. This accounted for the significant difference in publishing impactful articles between Chinese and American authors largely (Table 1).

Collaboration can enhance the power, efficiency, generalizability, and rapid completion of clinical research (21), and hence may improve the research quality large probably. Over the past 5 years, for instance, all 17 randomized controlled trials searched for “sepsis” in the New England Journal of Medicine were interagency collaborations. In addition, only one out of the top 10 most frequently cited articles in the field of CCM was written by Nusbaum independently (9). Significantly, the success of clinical trial groups such as the Canadian Critical Care Trials Group (CCCTG) (22) and Australia and New Zealand Intensive Care Society Clinical Trials Group (ANZICS-CTG) (23) has fueled efforts to build similar collaboration models around the world. In China, an investigator-led group, China Critical Care Clinical Trials Group (CCCCTG) was launched 20 years ago (24), and was active in Critical Care researches over the ensuing years. By this bibliometric analysis, however, it was revealed that collaborations between either domestic or international authors were limited in CCM researches. Moreover, the results showed that the most frequent collaborations took place among the authors who served in the same ICU (Figure 4, Supplementary Table S7). Therefore, collaboration could be a modifiable factor to promote the research quality of Chinese intensivists in future.

Funding is important to facilitate either basic or clinical medical research. However, CCM research not only in China, but around the world, was under-funded in comparison with other specialties, although critical illnesses became a burden of healthcare increasingly. According to Coopersmith's report, 332 (1.7%) out of 19,257 grants funded by the National Institutes of Health were definitely related to critical care and a maximum of 1,212 (6.3%) grants were possibly related to critical care (25). It was demonstrated that 5,624 (41.6%) out of 13,487 publications reported funding in this study. Additionally, we performed a search on the Website Science net (https://fund.sciencenet.cn/) for grants from catalog of H15 (“acute and intensive care medicine/trauma/burns/plastic surgery”) of NSFC (National Natural Science Foundation of China) and successfully applied by the Chinese intensivists from 1 January 2016 to 31 December 2020. Of a total of 1,073 (517.85 million RMB Yuan) funded projects, as shown in Supplementary Table S8, only 141 (13.14%; 6.344 million out of 517.85 million RMB Yuan) led by Chinese intensivists have been approved. Interestingly, rapid growth in clinical trials was found in both websites Clinical Trials (https://clinicaltrials.gov/) and ChiCTR (Chinese Clinical Trail Registry, http://www.chictr.org.cn) registered by Chinese intensivists from 1 January 2016 to 31 December 2020 (Supplementary Figure S5). These findings suggested that multiple resources of funding would be a possible strategy to promote Chinese CCM research in future.

There were several limitations in this study. First, this research was only based on the electronic database of the Web of Science, while other electronic databases were not searched and analyzed, such as PubMed, Embase, and Cochrane Library, especially published in Chinese Literature databases such as CNKI, CQVIP, Wanfang, etc. Second, there were some flaws in our data source. For example, an author signed different names of hospital / institute / university in his / her different published articles, making the system unable to identify the articles published by the same person. Third, the software defaults so that the acronym cannot be changed. and if you want to change it, you may need to do the later stage of photoshop (but this may cause manual revision and non-repeatability of the results). Fourth, there may be differences in data recognition by different software, resulting in possible errors in results. Finally, when calculating clinical registration research items, we cannot completely exclude a very small number of projects led by respiratory and critical illness experts, anesthesiologists, or other emergency department experts from being included in this study.

## Conclusion

This bibliometric analysis demonstrated that CCM research in China grew rapidly in recent 20 years. However, the impactful publications remained limited. The results of this study suggested that the lack of universality, as well as a comprehensive training in scientific researches, inactive collaboration, and underfunded, be the important barriers to the promotion of the quality and quantity of Chinese CCM research.

## Data availability statement

The original contributions presented in the study are included in the article/Supplementary material, further inquiries can be directed to the corresponding author.

## Author contributions

WQ, CX, and ZL contributed to the acquisition, analysis, interpretation of data, statistical analysis, data arrangement, and draft of the manuscript. LY contributed to interpret the results. FS contributed to supervision manuscript. LZ completed all statistical analyses of this study. PM contributed to study concept, supervision, organize the final manuscript, identified as the guarantor of the article, taking responsibility for the integrity of the work, and from inception to published article. All authors contributed to the article and approved the submitted version.

## Conflict of interest

The authors declare that the research was conducted in the absence of any commercial or financial relationships that could be construed as a potential conflict of interest.

## Publisher's note

All claims expressed in this article are solely those of the authors and do not necessarily represent those of their affiliated organizations, or those of the publisher, the editors and the reviewers. Any product that may be evaluated in this article, or claim that may be made by its manufacturer, is not guaranteed or endorsed by the publisher.
