# Supplementary material for: Impactful publications of critical care medicine research in China: A bibliometric analysis
Source: Front Med (Lausanne). 2022 Oct 18;9:974025. doi: 10.3389/fmed.2022.974025 (PMC9622943; doi:10.3389/fmed.2022.974025)
Supplement: Supplementary file 1 [file Data_Sheet_1.pdf]

Table S1 The proportion of publications by Chinese and American authors

Year	Total	Chinese authors		American authors	
		N	Proportion (%)	N	Proportion (%)
2001	1547	5	0.32	569	36.78
2002	1662	7	0.42	617	37.12
2003	1839	3	0.16	606	32.95
2004	1884	8	0.42	623	33.07
2005	2039	5	0.25	653	32.03
2006	2533	10	0.39	857	33.83
2007	2627	13	0.49	811	30.87
2008	2820	14	0.50	921	32.66
2009	3269	42	1.28	920	28.14
2010	3502	58	1.66	941	26.87
2011	3732	77	2.06	1119	29.98
2012	4227	109	2.58	1158	27.40
2013	4458	242	5.43	1167	26.18
2014	4925	331	6.72	1332	27.05
2015	5293	475	8.97	1357	25.64
2016	6087	631	10.37	1556	25.56
2017	6616	774	11.70	1672	25.27
2018	7536	925	12.27	1808	23.99
2019	8473	1169	13.80	1936	22.85
2020	10692	1724	16.12	2196	20.54
Total	85761	6622	1.85(0.42,10.01) #	22819	27.77(25.58,32.88) #

N：numbers；#Median (interquartile range,(IQR))

Table S2 Publications (The number/the total) in top 10 impactful journals in the field of Critical Care Medicine by Chinese versus America intensivists

Journal	IF (2020)	By Chinese authors (total=6622)		By American authors （total=22819）		
		Number	%	Number	%	*P*-value
NEJM	91.25	0	0.00	56	0.245	<0.001
The Lancet	79.32	5	0.08	24	0.11	0.489
JAMA	56.27	5	0.08	160	0.70	<0.001
BMJ	39.89	0	0.65	19	0.08	<0.001
**Publications in the top 4 journals**	**--**	**53**	**0.80**	**263**	**1.15**	**0.014**
Am J Resp Crit Care Med	21.40	7	0.11	317	1.39	<0.001
Intensive Care Med	17.44	66	0.99	208	0.91	0.525
CHEST	9.41	26	0.39	228	1.00	<0.001
Critical Care	9.10	167	2.52	331	1.45	<0.001
Critical Care Med	7.60	62	0.94	1701	7.45	<0.001
Ann Intensive Care	6.93	20	0.30	16	0.07	<0.001
**Publications in the top 10 journals**	**--**	**358**	**5.41**	**3060**	**13.41**	**<0.001**

NEJM (NEW ENGLAND JOURNAL OF MEDICINE); JAMA (JOURNAL OF THE AMERICAN MEDICAL ASSOCIATION); BMJ (British Medical Journal); Am J Resp Crit Care Med (AMERICAN JOURNAL OF RESPIRATORY AND CRITICAL CARE MEDICINE); Intensive Care Med（INTENSIVE CARE MEDICINE）; Critical Care Med (CRITICAL CARE MEDICINE; Ann Intensive Care (ANNALS OF INTENSIVE CARE).

Table S3 Top 20 highly co-occurrenced keywords in publications by Chinese versus American authors from 2001 to 2020

	By Chinese authors		By American authors	
Rank.	keywords	occurrences	keyword	occurrences
1	sepsis	642	pediatric	1402
2	ALI	412	trauma	624
3	inflammation	289	sepsis	605
4	apoptosis	268	CC	584
5	mortality	232	ICU	546
6	meta-analysis	214	mortality	448
7	prognosis	199	traumatic brain injury	372
8	AKI	175	cardiac arrest	328
9	lipopolysaccharide	138	ALI	302
10	ICU	124	outcomes	294
11	septic shock	120	CPR	290
12	oxidative stress	114	MV	273
13	covid-19	95	inflammation	259
14	MV	87	ECMO	226
15	NF-KB	85	fluid resuscitation	225
16	autophagy	77	AKI	215
17	risk factors	71	critical illness	191
18	biomarker	62	outcome	183
19	dexmedetomidine	58	septic shock	180
20	proliferation	57	stroke	176

Occurrences were calculated on co-occurrence frequency of the keywords in articles by Chinese or American intensivists. ALI: acute lung injury; AKI: Acute kidney injury; ECMO: extracorporeal membrane oxygenation ICU: intensive care unit; CPR: cardiopulmonary resuscitation; COVID-19: coronavirus disease 2019; MV: mechanical ventilation; CC: critical care; NF-KB: NF-kappab;

Table S4 Top10 earliest and latest keywords by time trends on Critical Care Medicine articles by Chinese versus American authors from 2001 to 2020

		Chinese		America	
	Rank.	keyword	Avg.pub.year	keyword	Avg.pub.year
Earliest	1	dobutamine	2007.17	NF-kB	2005.44
	2	ACS	2011.67	cost-effectiveness	2005.60
	3	hospital mortality	2012.88	TNF-alpha	2005.92
	4	dopamine	2013.00	reperfusion	2006.10
	5	IAH	2013.00	lipopolysaccharide	2006.26
	6	IRI	2013.75	activated protein c	2006.57
	7	pain	2014.00	defibrillation	2006.82
	8	NF-kB	2014.29	hemofiltration	2006.86
	9	complication	2014.33	human	2006.97
	10	escherichia coli	2014.33	abuse liability	2007.20
Latest	1	coronavirus disease 2019	2020.25	sars-cov-2	2020.21
	2	cytokine storm	2020.14	covid-19	2020.12
	3	covid-19	2020.03	coronavirus	2020.07
	4	sars-cov-2	2020.00	machine learning	2018.50
	5	coronavirus	2020.00	critical care outcomes	2018.41
	6	nomogram	2020.00	angiotensin ii	2018.38
	7	aging	2020.00	precision medicine	2018.17
	8	2019-ncov	2020.00	global health	2018.14
	9	coordination polymer	2020.00	TTM	2017.94
	10	severity	2019.88	pediatric ARDS	2017.94

Avg.pub.year: the average publication year of the articles in which the keyword occurs (to the nearest 2 decimal place). ACS: abdominal compartment syndrome; COVID-19: coronavirus disease 2019; IAH: intra-abdominal hypertension; NF-kB: nuclear factor-kappa b; TNF-alpha: tumor necrosis factor-alpha; IRI: ischemia/reperfusion injury; TTM: targeted temperature management; ARDS: acute respiratory distress syndrome

Table S5 The top ten highly cited articles from 2001 to 2020（China and World）

Published by Chinse authors				Published by authors in the world			
Authors (year)	Title	Journal (IF-2020)	Citations	Authors (year)	Title	Journal (IF-2020)	Citations
Yang, XB; et al. (2020)	Clinical course and outcomes of critically ill patients with SARS-CoV-2 pneumonia in Wuhan, China: a single-centered, retrospective, observational study*#	LANCET RESPIRATORY MEDICINE (30.7)	3753	Liberati, A; et al. (2009)	The PRISMA Statement for Reporting Systematic Reviews and Meta-Analyses of Studies That Evaluate Health Care Interventions: Explanation and Elaboration	ANNALS OF INTERNAL MEDICINE (25.391)	10788
Li, H; et al. (2020)	Coronavirus disease 2019 (COVID-19): current status and future perspectives*#	INTERNATIONAL JOURNAL OF ANTIMICROBIAL AGENTS (5.283)	435	Lim, SS; et al. (2012)	A comparative risk assessment of burden of disease and injury attributable to 67 risk factors and risk factor clusters in 21 regions, 1990-2010: a systematic analysis for the Global Burden of Disease Study 2010*	LANCET (79.323)	7333
Liu, JY; et al. (2020)	Neutrophil-to-lymphocyte ratio predicts critical illness patients with 2019 coronavirus disease in the early stage*	JOURNAL OF TRANSLATIONAL MEDICINE (5.53)	437	Mehta, RL; et al. (2007)	Acute Kidney Injury Network: report of an initiative to improve outcomes in acute kidney injury	CRITICAL CARE (9.097)	4971
Peng, QY; et al. (2020)	Findings of lung ultrasonography of novel corona virus pneumonia during the 2019-2020 epidemic	INTENSIVE CARE MEDICINE (17.44)	425	Bellomo,R; et al. (2004)	Acute renal failure - definition, outcome measures, animal models, fluid therapy and information technology needs: the Second International Consensus Conference of the Acute Dialysis Quality Initiative (ADQI) Group	CRITICAL CARE (9.097)	4819
Su, X; et al. (2016)	Dexmedetomidine for prevention of delirium in elderly patients after non-cardiac surgery: a randomised, double-blind, placebo-controlled trial*	LANCET (79.323)	327	Levy, MM; et al. (2003)	2001 SCCM/ESICM/ACCP/ATS/SIS International Sepsis Definitions Conference	CRITICAL CARE MEDICINE (7.598)	4719

Mo,YY; et al. (2020)	Work stress among Chinese nurses to support Wuhan in fighting against COVID-19 epidemic*	JOURNAL OF NURSING MANAGEMENT (3.325)	300	Yang, XB; et al. (2020)	Clinical course and outcomes of critically ill patients with SARS-CoV-2 pneumonia in Wuhan, China: a single-centered, retrospective, observational study*#	LANCET RESPIRATORY MEDICINE (30.7)	3753
Zhang, S; et al. (2020)	Estimation of the reproductive number of novel coronavirus (COVID-19) and the probable outbreak size on the Diamond Princess cruise ship: A data-driven analysis*	INTERNATIONAL JOURNAL OF INFECTIOUS DISEASES (3.623)	308	Bernard, SA; et al. (2002)	Treatment of comatose survivors of out-of-hospital cardiac arrest with induced hypothermia	NEW ENGLAND JOURNAL OF MEDICINE (91.253)	3840
Lei L; et al. (2020)	Comparison of Prevalence and Associated Factors of Anxiety and Depression Among People Affected by versus People Unaffected by Quarantine During the COVID-9 Epidemic in Southwestern China*	MEDICAL SCIENCE MONITOR (2.649)	290	Rhodes, A et al. (2017)	Surviving Sepsis Campaign: International Guidelines for Management of Sepsis and Septic Shock: 2016*	CRITICAL CARE MEDICINE (7.598)	3388
Wang, L. (2020)	C-reactive protein levels in the early stage of COVID-19*	MEDECINE ET MALADIES INFECTIEUSES (2.152)	281	Finfer, S; et al. (2009)	Intensive versus Conventional Glucose Control in Critically Ill Patients	NEW ENGLAND JOURNAL OF MEDICINE (91.253)	3116
Su, LJ; et al. (2019)	Reactive Oxygen Species-Induced Lipid Peroxidation in Apoptosis, Autophagy, and Ferroptosis*	OXIDATIVE MEDICINE AND CELLULAR LONGEVITY (6.543)	278	Giugliano, RP; et al. (2013)	Edoxaban versus Warfarin in Patients with Atrial Fibrillation*	NEW ENGLAND JOURNAL OF MEDICINE (91.253)	3063

* highly cited paper : the papers of citations received by the top 1% of papers from each of 10 database years; # hot paper: the papers of citations received during the most recent two-month period by the top 0.1% of papers from the past two years.

Table S6 Geographical Distribution of the proportions of publications stratified with IF<5, IF⩾5 and IF⩾10 by Chinese authors

Province/City	Total	IF<5		IF⩾5		IF⩾10		*P*-value
		N	%	N	%	N	%	
BEIJING	1021	621	0.61	400	0.39	157	0.15	
ZHEJIANG	971	691	0.71	280	0.29	91	0.09	
SHANGHAI	779	504	0.65	274	0.35	86	0.11	
GUANGDONG	782	519	0.66	263	0.34	86	0.11	
JIANGSU	740	504	0.68	236	0.32	81	0.11	
HUBEI	520	312	0.60	208	0.40	52	0.10	
SICHUAN	389	251	0.65	138	0.35	44	0.11	
SHANDONG	643	516	0.80	118	0.18	19	0.03	
HUNAN	260	168	0.65	92	0.36	26	0.10	
HENAN	235	152	0.65	83	0.36	27	0.11	
HEILONGJIANG	201	138	0.69	63	0.31	19	0.09	
FUJIAN	169	107	0.63	62	0.37	25	0.15	
CHONGQING	152	102	0.67	50	0.33	16	0.11	
LIAONING	211	161	0.76	50	0.24	16	0.08	<0.001
JILIN	139	101	0.73	38	0.27	9	0.06	
TIANJIN	148	110	0.74	38	0.26	7	0.047	
ANHUI	124	90	0.73	34	0.27	11	0.08	
HEBEI	98	70	0.71	28	0.29	9	0.09	
GUIZHOU	83	56	0.67	27	0.33	7	0.08	
SHAANXI	103	76	0.74	27	0.26	5	0.05	
JIANGXI	89	69	0.78	20	0.22	8	0.09	
YUNNAN	57	41	0.72	16	0.28	2	0.04	
GUANGXI	69	54	0.78	15	0.22	7	0.10	
GANSU	82	68	0.83	14	0.17	4	0.05	
HAINAN	42	28	0.67	14	0.33	2	0.05	
NINGXIA	26	14	0.54	12	0.46	2	0.08	
XINJIANG	65	53	0.85	12	0.18	2	0.03	
INNER MONGOLIA	35	25	0.71	10	0.29	2	0.06	
SHANXI	26	20	0.77	6	0.23	0	0	
QINGHAI	15	10	0.67	5	0.33	3	0.20	
TIBET	3	1	0.33	2	0.67	0	0	

N： numbers

Table S7 Top 10 authors/ institutes/ countries ranked by the total collaboration link strength*

Rank	author	total link strength	N	institute	total link strength	N	country	total link strength	N
1	yang, yi	419	143	capital med univ	1162	744	peoples r china	5034	12698
2	qiu, haibo	413	126	china med univ	1072	422	usa	3581	1954
3	liu, ling	273	76	shanghai jiao tong univ	1037	679	italy	1410	208
4	liu, dawei	271	107	huazhong univ sci & technol	787	474	england	1251	287
5	yuan, chun-su	217	89	sichuan univ	770	619	canada	1248	286
6	wang, chong-zhi	217	88	fudan univ	760	515	australia	1224	243
7	long, yun	211	73	zhejiang univ	748	809	germany	1160	194
8	liu, jin	195	119	chinese acad med sci	745	398	netherlands	1016	161
9	tang, wanchun	194	100	sun yat sen univ	731	577	japan	899	189
10	wang, hao	178	92	southern med univ	718	371	france	864	135

N:numbers, *The total link strength was calculated on the number of publications co-authored by the authors, institutes and countries.

Table S8 The number and the grant amount of National Natural Science Foundation of China (NSFC) led by Chinese intensivists from 2016 to 2020

Year	Number of funded projects	Amount of Funding (RMB Yuan)
2016	11	590,000
2017	19	703,000
2018	24	931,000
2019	47	2,026,000
2020	40	2,094,000

RMB: Renminbi

Figure S1.
Pie chart of proportion of publications based on journal's impact factors (IF) by first or corresponding authors with affiliations of Critical Care Medicine in China from 2001 to 2020.
The color of palate represented the journal's IF of the articles, marked on red (IF≥10), black (IF >5, but IF<10) ,grey (IF≤5) and white (without IF) as well.

Figure S2
The top 10 countries in the world ranked by publications on CCM research
The X-axis represented each year from 2001 to 2020, the Y-axis represented the rank and the proportion of publication by authors from affiliations of CCM department or ICU in hospitals of the top 10 countries in the world ranked by the number of annual publications. The part of bar marked on black represented the proportion of publications by intensivists from America, and the red one represented the proportion of publications by intensivists of China. The parts from top to bottom of the figure represented the order ranking first to tenth.

Figure S3.
Visualization map of keywords overlay analysis on Critical Care Medicine articles published by Chinese (S-3A)vs American (S-3B) intensivists from 2001 to 2020.
The size of the circles indicated the co-occurrence frequency of keywords. The color indicated average publication year of the articles in which the keyword occurs,
the connecting lines indicated co-occurrence of the 2 keywords at both ends, the thickness of lines between circles indicated strength of linkage calculate on the frequency of co-occurrence.

Figure S4.
Pie chart of organization type based on institutes of the first and corresponding authors from 2001 to 2020.
Academic institutes：Primary function was education, degrees awarded. Name contains University, College, URL ends in .edu.
Medical research institutes that were part of an academic organization should be Academic (e.g., Sichuan University).
Research institutes：Organizations that were primarily focused on research (e.g., CHINESE ACAD MED SCI)
Non-academic hospitals：Hospitals, Medical Centers, organizations that treated or diagnosed patients (e.g., BEIJING HOSPITAL)
Others：Business, legal entity had a board of directors, a stock symbol, URL ends in .com (e.g., China National Pharmaceutical Corporation).
Government agencies：State owned or operated organizations. Name contained Municipal, National, they report to a Ministry, State or Federal Government funded, URL ends in .gov or country code such as .de, .ca, or .ch (e.g., Ministry of Education, China).

Figure S5.
Trials registered by Chinese authors with institutes of Critical Care Medicine from 2016 to 2020.
The X-axis represented the year, the Y-axis represented the number of projects. The clinical research projects registered in ClinicalTrials.gov were represented solid line. The clinical research projects registered in chictr.org.cn were represented dotted line. Clinical Trials: Projects registered in https://clinicaltrials.gov/. Chictr: Projects registered in https://www.chictr.org.cn.
