# Supplementary figures and images for: Impactful publications of critical care medicine research in China: A bibliometric analysis
Source: Front Med (Lausanne). 2022 Oct 18;9:974025. doi: 10.3389/fmed.2022.974025 (PMC9622943; doi:10.3389/fmed.2022.974025)

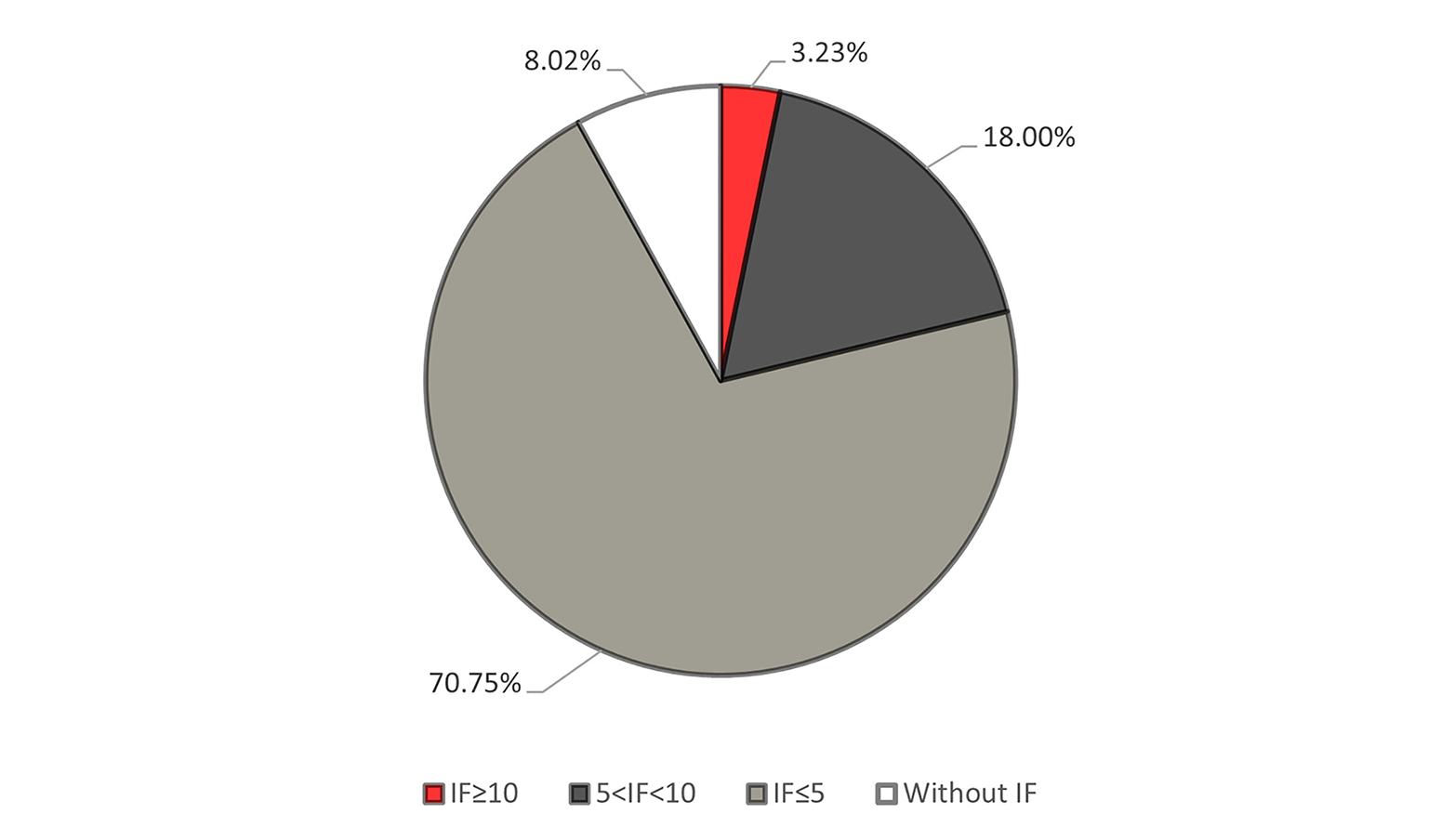

Supplement: Supplementary file 2 [file Image_1.TIF]

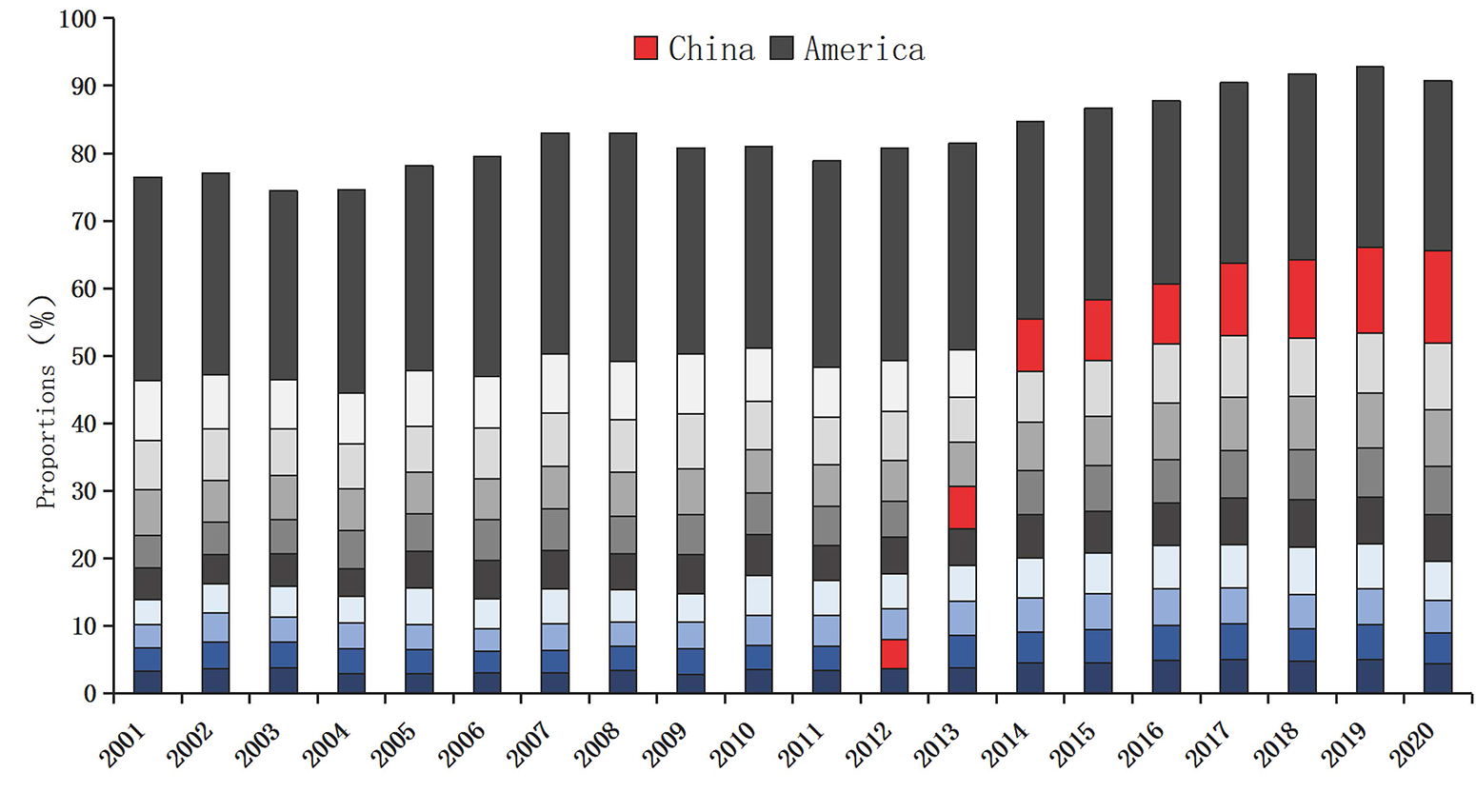

Supplement: Supplementary file 3 [file Image_2.TIF]

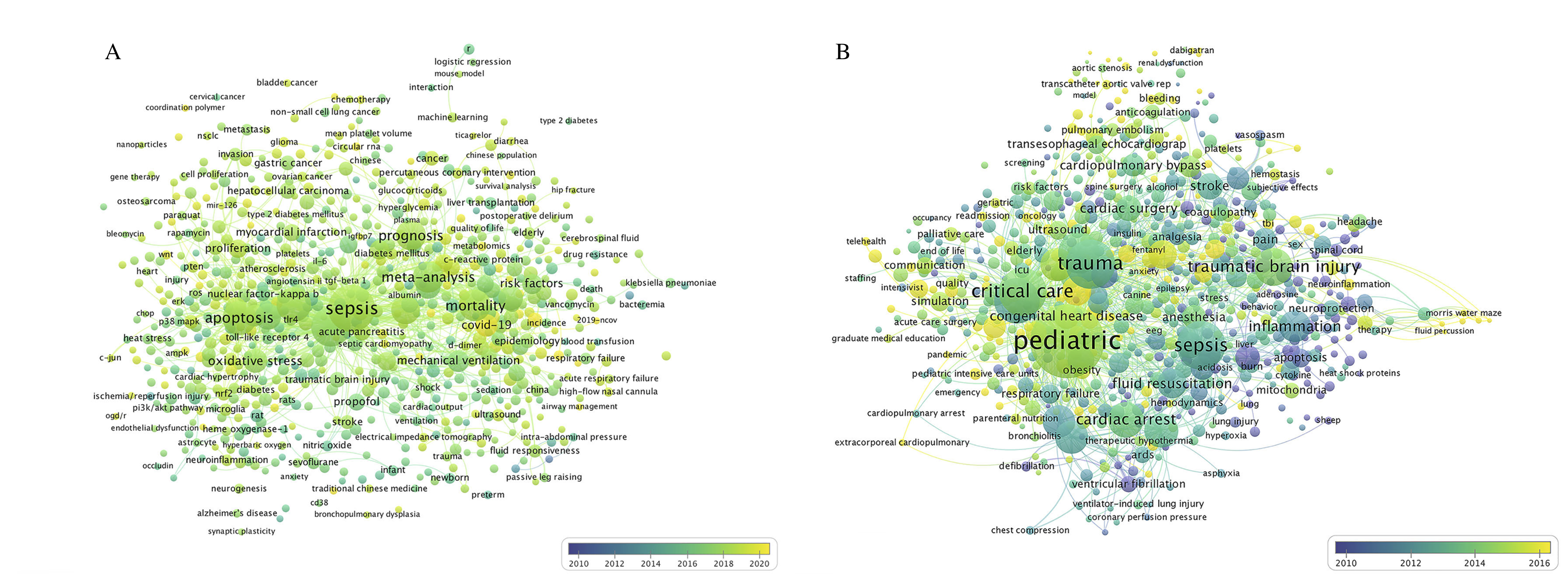

Supplement: Supplementary file 4 [file Image_3.TIF]

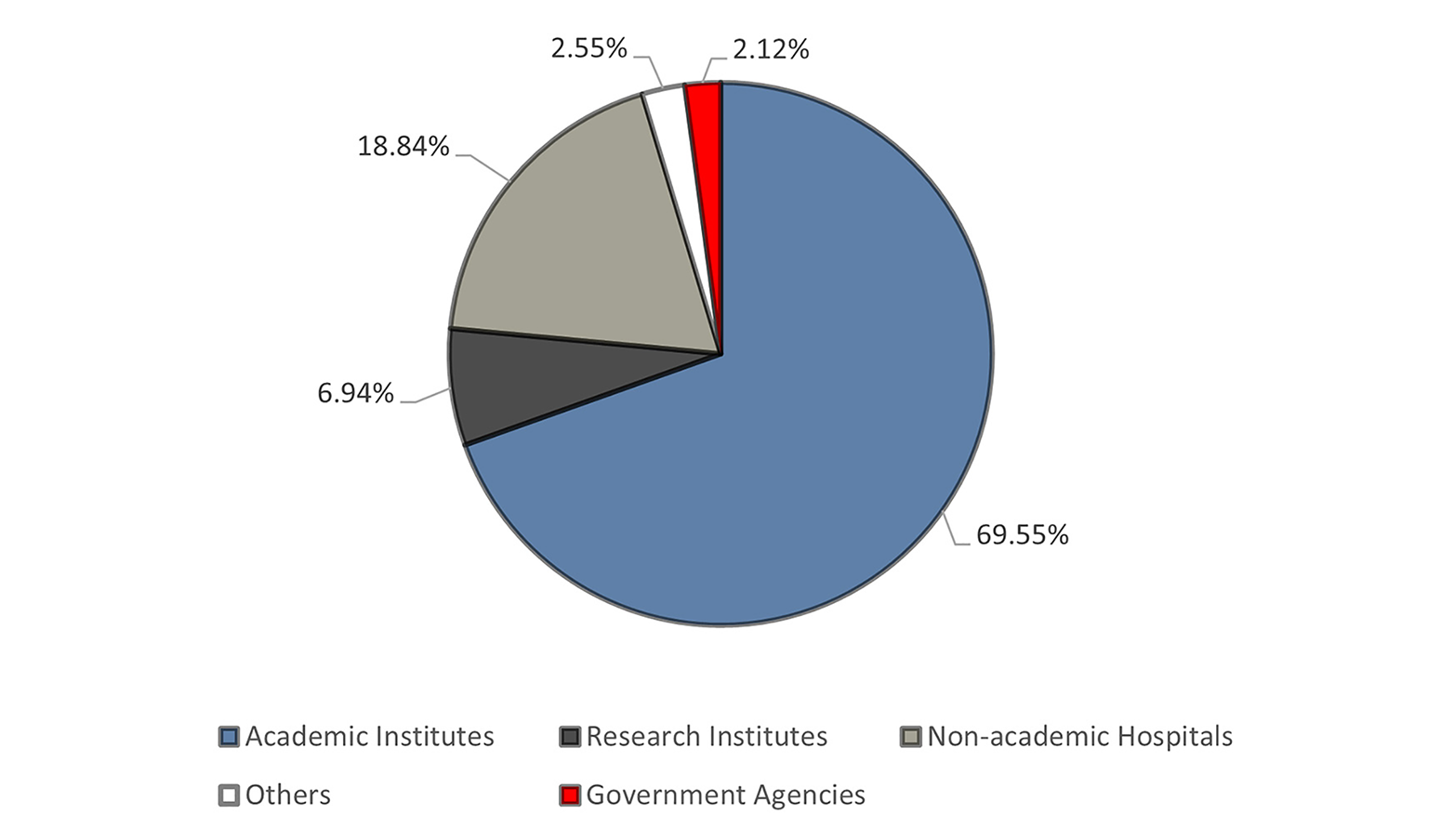

Supplement: Supplementary file 5 [file Image_4.TIF]

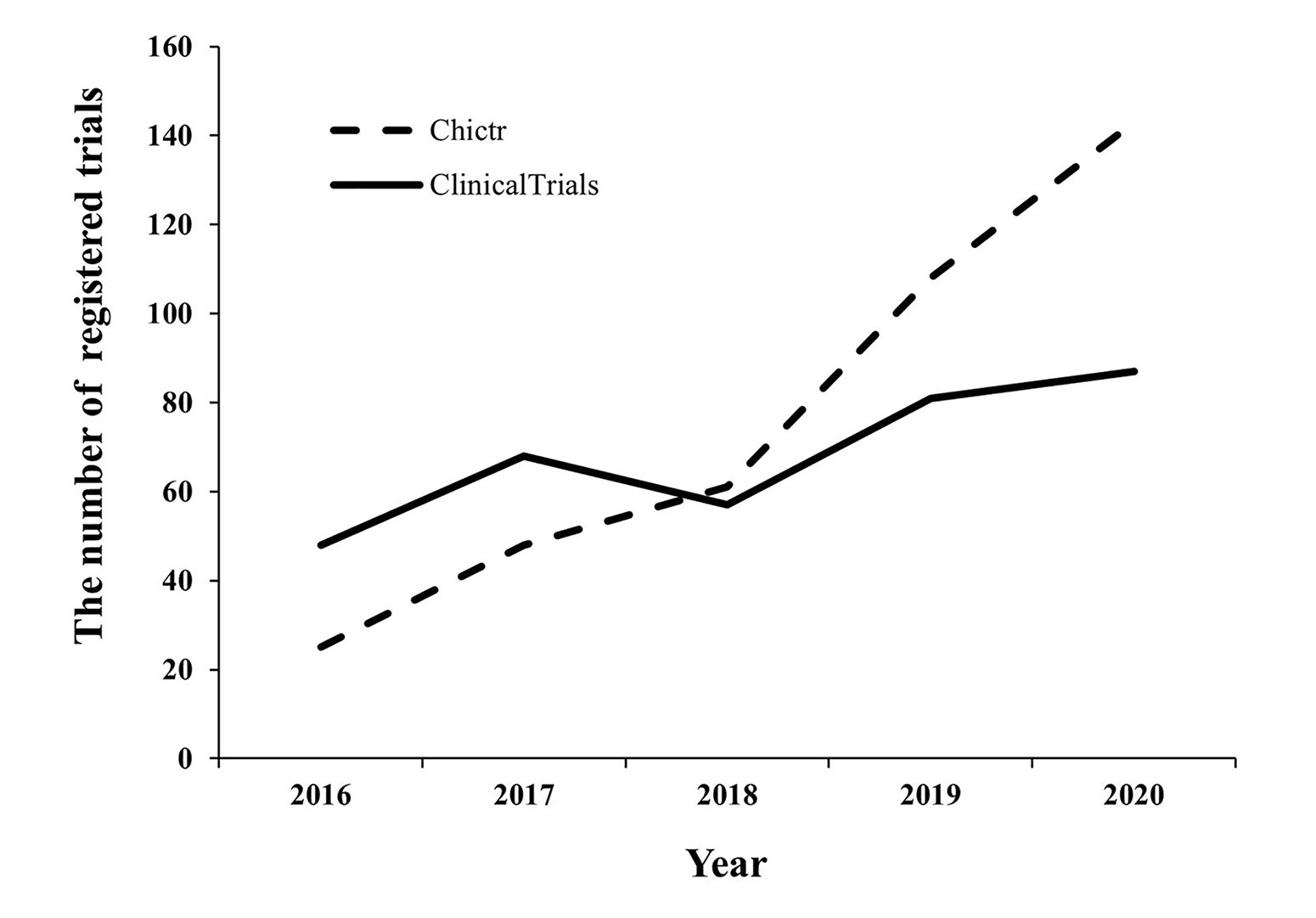

Supplement: Supplementary file 6 [file Image_5.TIF]
